# Quantitative Analysis of Genetic and Neuronal Multi-Perturbation Experiments

**DOI:** 10.1371/journal.pcbi.0010064

**Published:** 2005-11-25

**Authors:** Alon Kaufman, Alon Keinan, Isaac Meilijson, Martin Kupiec, Eytan Ruppin

**Affiliations:** 1 Interdisciplinary Center for Neural Computation, Hebrew University, Jerusalem, Israel; 2 School of Computer Science, Tel-Aviv University, Tel-Aviv, Israel; 3 School of Mathematical Sciences, Tel-Aviv University, Tel-Aviv, Israel; 4 Department of Molecular Microbiology and Biotechnology, Tel-Aviv University, Tel-Aviv, Israel; 5 School of Medicine, Tel-Aviv University, Tel-Aviv, Israel; Memorial Sloan Kettering Cancer Center, United States of America

## Abstract

Perturbation studies, in which functional performance is measured after deletion, mutation, or lesion of elements of a biological system, have been traditionally employed in many fields in biology. The vast majority of these studies have been qualitative and have employed single perturbations, often resulting in little phenotypic effect. Recently, newly emerging experimental techniques have allowed researchers to carry out concomitant multi-perturbations and to uncover the causal functional contributions of system elements. This study presents a rigorous and quantitative multi-perturbation analysis of gene knockout and neuronal ablation experiments. In both cases, a quantification of the elements' contributions, and new insights and predictions, are provided. Multi-perturbation analysis has a potentially wide range of applications and is gradually becoming an essential tool in biology.

## Synopsis

Which are the important elements of a system? What are their relative contributions to the performance of the various tasks the system is involved in? These simple and basic questions typically arise when analyzing the workings of any system, and of biological systems in particular. In the latter, the elements may be genes, proteins, cells, or tissues, depending on the level and scope of the analysis. To address these questions in a causal manner, perturbations are required, where the elements are perturbed and the resulting performance function is recorded. This approach has been one of the cornerstones of biological research. However, it has usually been confined to the perturbation of a single element at a time, which may lead to misleading results if the elements of the system functionally interact with each other. This paper addresses these questions by providing a quantitative and rigorous method for the analysis of multi-perturbation experiments, where more than one element may be concomitantly perturbed. The workings of the new method are demonstrated in the analysis of genetic multi-knockout experiments of DNA repair in the yeast *Saccharomyces cerevisiae* and a neural circuit in the nematode *Caenorhabditis elegans* accounting for chemotaxis. However, the method is general and can be applied to study many other systems in numerous pertinent biological domains.

## Introduction

System identification (localization of function) in biological networks is currently mainly studied in genetics by high-throughput expression profiling and in neuroscience by functional brain imaging. While these techniques have proved to be very useful and productive [1,2], the correlation-based approach they employ does not necessarily identify causal relations. Previous studies have shown that there may be, at times, a weak correlation between the expression of different genes and their role in various cellular functions [3,4]. In a similar vein, the need to add causal perturbation analysis to complement correlation-based ones has also been raised in the neuroscience literature (e.g., [5,6]). To causally deduce the roles played by system elements (genes, proteins, neurons, brain regions, etc.), perturbation studies, in which functional performance is measured after deletion, mutation, or lesion of the different elements, have traditionally been employed. However, the vast majority of these studies have perturbed only one element at a time, often resulting in little phenotypic effect. Hence, in complex biological systems, multiple concomitant perturbations should be employed to reveal the contributions of the different elements to the system's functioning. In genetics, the lack of phenotypic effects may be due to the existence of duplicates, alternative pathways, and functional overlaps [1,7]. To uncover these effects, new experimental techniques are now emerging to carry out the necessary multi-perturbation studies [1,8,9]. Specifically, the recent discovery of RNA interference [10,11] and the rapid recent advances in gene silencing with RNA interference chips [12,13] may advance multi-perturbation technology to our doorstep. In neuroscience, techniques such as transcranial magnetic stimulation allow researchers to induce reversible “virtual lesions,” enabling them to perform multi-lesion experiments for the analysis of cognitive and perceptual tasks in humans [14,15]. A question remains: how can the results of such multi-perturbation experiments be integrated and analyzed, and what knowledge can be extracted from them?

To address this challenge, Keinan et al. [16] developed the multi-perturbation Shapley value analysis (MPA) method, and presented its application to the analysis of a neural model of the lamprey swimming controller and to the analysis of reversible cooling deactivation experiments in cats. Here we expand these results in two fundamental ways. First, we present the first application of the MPA to the analysis of gene knockout experiments and to the analysis of neuronal ablation data. Second, we present a complementary method for the analysis of multi-perturbation data, the functional influence network (FIN) algorithm. In contrast to existing methods for network inference and system identification in biology—which employ methods from machine learning such as Bayesian network inference [17,18], Boolean networks [19], and other reverse engineering methods [20]—both MPA and FIN are based on concepts from game theory. These new methods are the first to utilize fundamental results from game theory to assess the contribution of system elements and functional subsets of such elements to the overall system performance function. For the task of determining the functional contribution of system elements, these game theory tools are more adequate than standard machine learning approaches employing error minimization (e.g., [21]), since they are based on a solid axiomatic framework and provide a unique contribution assignment [16]. Recently, there have been a number of attempts to utilize game theory approaches in neuroscience, but these had a completely different goal of constructing decision-making models [22,23].

The goal of MPA is to define and calculate the contribution (importance) of system elements to a certain function, from a dataset of a series of multi-perturbation experiments. In each such experiment, a different subset of the system elements is concomitantly perturbed (denoting a perturbation configuration), and the system's performance in the studied function is measured. The FIN algorithm analyzes the same multi-perturbation data. It describes the incremental contribution of each subset of elements to the function studied, and produces a compact representation, composed only of the most important subsets. As the full set of all theoretically possible multi-perturbation experiments required for the MPA and FIN computation is usually unavailable, both analyses employ a predictor algorithm to compute the system's performance on the missing multi-perturbation experiments.

In the following sections we describe the MPA and FIN methods and present their application to two different biological systems: the DNA post-replication repair (PRR) pathway in *Saccharomyces cerevisiae,* and laser ablation studies of *Caenorhabditis elegans* chemosensory neurons.

## Results

### Quantitative Multi-Perturbation Analysis

#### Multi-perturbation Shapley value analysis.

The starting point of MPA [16] is a dataset of multi-perturbation experiments studying a system's performance in a certain function. In each such experiment, a different subset of the system's elements are perturbed concomitantly (denoting a perturbation configuration), and the system's performance following the perturbation is measured. Given this dataset, the goal of MPA is to ascribe to each element its contribution (importance) to the studied function.

The basic observation underlying MPA is that the multi-perturbation setup is essentially equivalent to a coalitional game. A coalitional game is defined by a pair (*N, v*), where *N =* {1,…, *n*} is the set of all players and *v*(*S*), for every *S* ⊆ *N,* is a real number associating a worth with the coalition *S,* and *v*(∅︀) = 0. In the context of multi-perturbations, *N* denotes the set of all the system's elements, and for each *S* ⊆ *N, v*(*S*) denotes the performance measured under the perturbation configuration in which all the elements in *S* are intact and the rest are perturbed.

A payoff profile of a coalitional game is the assignment of a payoff to each of the players. A value is a function that assigns a unique payoff profile to a coalitional game. The function is efficient if the sum of the payoffs assigned to all players is *v*(*N*). The definite efficient value in game theory and economics for coalitional games is the Shapley value [24], defined as follows: let the marginal importance of player *i* to a coalition *S,* with *i* ∉ *S,* be


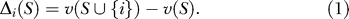


Then, the Shapley value is defined by the payoff


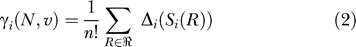


assigned to player *i,* for all *i* ∈ *N,* where ℜ is the set of all *n*! orderings of *N,* and *S_i_*(*R*) is the set of players preceding *i* in the ordering *R*. The Shapley value has a clear intuitive interpretation, denoting the average marginal importance of player *i* to the game. Importantly, it has an axiomatic foundation (see Text S1), which is well suited for the analysis of biological data [16]. The MPA uses the Shapley value as the unique fair measure of each element's contribution (importance) to the function in question.

Obviously, conducting the large number of multi-perturbation experiments (exponential to the size of the system) required for the computation of the Shapley value is most often intractable. In such cases, MPA involves training a predictor using a given subset of multi-perturbation experiments to predict the performance levels of all missing experiments. Given the predicted outcomes of all multi-perturbation experiments, the predicted Shapley value is calculated as the Shapley value based on these outcomes. The accuracy of such an analysis depends on the accuracy of the predictions [16] and is determined using standard cross validation techniques such as leave-one-out [25]. The analysis presented throughout this paper is based on a projection pursuit regression predictor [26]. The accuracy of the predicted contributions is strongly dependent on the accuracy of the predictor used. However, the outcome—the predicted contributions—is more accurate then the individual predictions provided by the predictor because of the fact that the Shapley value is obtained via an averaging over a large number of predictions. Assuming that the predictor is unbiased, prediction errors tend to cancel each other out, resulting in a predicted Shapley value that is unbiased and very similar to the real one. See Protocol S1 for a detailed discussion on the robustness and stability of the predicted contributions produced by the MPA.

#### Functional influence network.

The FIN algorithm, based on the series of multi-perturbation experiments, begins with the computation of a performance prediction function *F,* in the form of a multilinear weighted summation over all 2*^n^* subsets (summands) of the *n* elements in the system (see Materials and Methods). The weight of each summand describes the marginal contribution of that subset of elements to the value of *F*. Given a configuration *S* of perturbed and intact elements, the goal of *F*(*S*) is to accurately describe the experimentally measured performance value of the system in the task studied under that configuration, *v*(*S*). After obtaining *F,* the FIN algorithm proceeds to prune its summands and retains only the most significant ones, to obtain a compact, approximate performance prediction function *F˜* (the detailed algorithm is provided in the Materials and Methods). The latter preserves a high percentage of *F'*s original prediction accuracy but aims to provide a compact functional description that may be visualized, if sufficiently compact. Each summand of *F* and *F˜* can be viewed as an integrative functional pathway in the sense that the knockout of any of its elements will zero its contribution. In cases where the full set of all possible multi-perturbation experiments required for the FIN computation is unavailable, the FIN, similar to MPA, uses a predictor algorithm to compute the system's performance in the missing experiments. The accuracy of the resulting FIN description for a given prediction accuracy is part of a broader conceptual issue, that of the relationship between “predictive knowledge” (the prediction accuracy) and “descriptive knowledge” (provided in the case in hand by the FIN output). A comprehensive investigation of this fundamental issue is out of the scope of the current work and will be addressed in a separate paper (preliminary results have been recently presented by Kaufman et al. in the BioPathways Special Interest Group, ISMB 2005).

### Gene Knockout Analysis: The Rad6 Pathway

#### Post-replication repair.

We performed a multi-knockout study of the DNA PRR system of the yeast *S. cerevisiae*. DNA repair pathways in yeast have been classified genetically into three major repair systems specialized on different types of damage: (1) the excision repair (Rad3) group, which is mainly involved in the repair of UV-irradiated DNA, (2) the recombination repair (Rad52) group, which is mainly involved in repair of damage caused by ionizing radiation and of double-strand breaks in the DNA, and (3) the PRR (Rad6) group of genes. This last pathway is believed to facilitate replication in situations where lesions in the template strand would otherwise cause a stalling of the replication machinery, as occurring following UV radiation.

A key physiological target of the PRR pathway is PCNA, a homotrimeric ring-shaped protein that encircles DNA, functioning as a freely sliding clamp that tethers DNA polymerase to the DNA template. The current hypothesis posits that following the stalling of the replicative DNA polymerases (when lesions are encountered), PCNA is modified, and the replicative polymerase is replaced by trans-lesion polymerases. Ubiquitination of PCNA is carried out by the Rad6 ubiquitin-conjugating enzyme, which is targeted to the stalled replication fork through physical interactions with the Rad18 cofactor [27,28]. During DNA synthesis, Replication factor C (RFC), a heteropentameric protein complex, is necessary for loading PCNA onto double-stranded DNA at the primer–template junction. Recently, several proteins with similarities to Rfc1 (the large subunit of RFC) were found to form RFC-like complexes (RLCs), including Elg1, Rad24, and Ctf18. These RLCs may act similarly to RFC, loading PCNA or PCNA-related complexes that act as clamps for specific DNA polymerases [29]. In addition to the three RLC genes, our study includes *RAD18,* a gene needed for PCNA modification, and *REV3,* which encodes an alternative DNA polymerase (ζ).

The analyzed data include a series of multi-knockout experiments carried out in the lab of one of the authors (M. K.), testing the ability of the resulting mutants to resolve the single-stranded gaps created after UV irradiation. Hence, the perturbations were gene knockouts, and the elements were the five genes listed above. The performance under investigation was UV survival, measured by the relative number of colonies that survived compared to the wild-type yeast strain (normalized on a scale from zero to one). The dataset included 21 multi-knockout experiments (see Table S1). Prediction of the full multi-knockout set (i.e., 32 multi-perturbations) was obtained using projection pursuit regression, and explains 79.6% of the data variance via leave-one-out cross validation.

#### MPA and FIN analysis.

**Figure 1:**
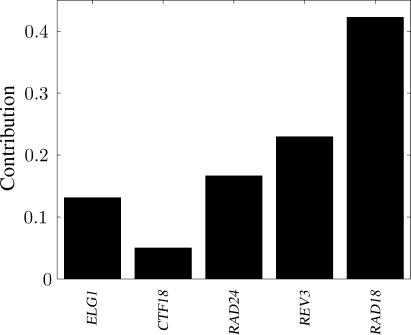
Contributions of Genes in the Rad6 Pathway to PRR Functioning (Normalized Such That Their Sum Equals One)

Figure 1 displays the results of MPA of the Rad6 data, leading to a quantification of the causal contribution of each of the Rad6 genes to PRR. The most important genes are *RAD18* and *REV3,* the modifier gene and the DNA polymerase, respectively. All three RLCs play a causal role as well, but their importance differs markedly.

**Figure 2:**
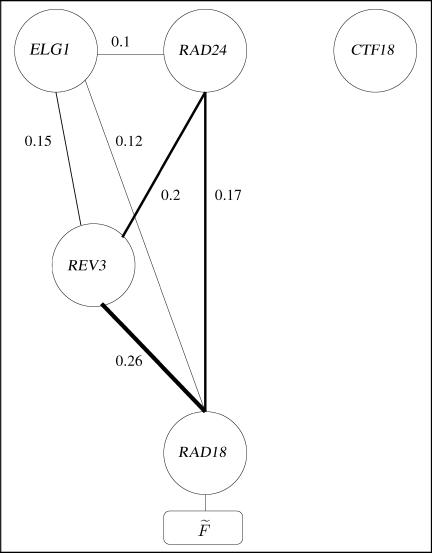
The FIN Diagram of the PRR Pathway Visualizes the compact performance function *F˜ = e ⋅ *(*d* ⋅ 0.26 + *c* ⋅ 0.17 + *a* ⋅ 0.12 + *d* ⋅ (*c* ⋅ 0.2 + *a* ⋅ 0.15) + (*c ⋅ a*) ⋅ 0.1), where *a* through *e* are Boolean variables representing the genes *(a = ELG1, b = CTF18 , c = RAD24 , d = REV3,* and *e = RAD18 )*. The investigated genes are represented as binary nodes whose state is determined according to the state of the corresponding genes in a given perturbation configuration, intact or knocked out. The nodes are connected with edges, their weight representing the functional influence between the two endpoint genes (the width of the edge is proportional to its weight). Given a knockout configuration, the expected performance level *F˜* can be calculated by summing up the weights on the edges between intact nodes that form a connected component with the function node *F˜*. For example, in a mutant where both *REV3* and *ELG1* are knocked-out, the intact nodes are *CTF18, RAD24,* and *RAD18*. The edge *RAD24*–*RAD18* is the resulting connected subgraph of *F˜* predicting a performance level of 0.17. Observe that there are three main (two-node) pathways leading to *F˜* *,* lines *RAD24*–*RAD18* , *ELG1*–*RAD18,* and *REV3*–*RAD18,* where *RAD18* is an essential gene in all of them. The RLC *CTF18* has no significance in the FIN description even though it has a contribution of 4% (see Figure 1); it contributes marginally across many insignificant summands and does not play a significant role in any major one.

The multi-knockout data can be utilized to construct a unique weighted multilinear performance prediction function *F,* which, given any configuration of knocked-out and intact genes, can accurately predict the PRR performance level. However, this function contains 32 (or, more generally, 2*^n^*) terms corresponding to all possible knockout configurations and hence is unintelligible and uninformative to the biologist. To extract the relevant information in the data and make it explicit, *F* is further processed via the FIN algorithm to construct a compact and yet fairly accurate functional prediction function, *F˜* . Each of the terms in *F˜* can be viewed as a serial functional pathway, whose contribution depends on the intactness of all its component genes. (Obviously, membership in a functional pathway does not necessarily imply that there are direct physical interactions between the elements of the pathway.) *F˜* 's compactness can be utilized to visualize the PRR functioning via a FIN diagram, as shown in Figure 2.

The FIN analysis gave rise to two new hypotheses. First, as is evident in Figure 2, both *ELG1* and *RAD24* play a significant role even without *REV3* (edges *RAD24*–*RAD18* and *ELG1*–*RAD18* in Figure 2). Hence, there is probably another polymerase (or perhaps more than one) involved in the PRR process, suggesting that both *ELG1* and *RAD24* play a role loading this additional DNA polymerase. A good candidate for such an additional polymerase is Pol-η, encoded by the *RAD30* gene. This alternative polymerase has been shown to be dependent on Rad6/Rad18 for activity. Second, the *REV3*–*RAD18* pathway (edge *REV3*–*RAD18* in Figure 2) encompasses 26% of the system's repair performance. This suggests that there are some additional DNA polymerase loaders besides those investigated. Alternatively, some of the functionality of the DNA polymerase may be maintained even in the absence of the RLCs.

### Analysis of Neuronal Ablations: Chemotaxis in *C. elegans*

We turn to address the question of function localization in the nervous system, focusing on laser ablation experiments of the *C. elegans* chemosensory neurons. The behavior studied in these experiments was chemotaxis, in which the nematode directs its movements according to chemical gradients in the environment, moving toward the highest concentration of food or fleeing from toxins. Reanalyzing the data published by Bargmann and Horvitz [30], we compared the qualitative conclusions given in their paper to the quantitative analogs obtained by applying the MPA. The elements studied were eight sensory neuron pairs (out of a total of 16 pairs that form the chemosensory system [31]). In each laser ablation experiment both neurons in a pair were either left intact or perturbed. The performance measures, chemotaxis to various attractants (each composing a distinct functional task), were evaluated by placing the animal on an agar plate with a gradient of an attractant on one side of the plate, and scoring the chemotaxis performance by counting the number of times the animal arrived at the peak of the gradient minus the number of times the animal arrived at the control plug at the opposite side of the plate. The level of chemotaxis performance was evaluated under 31 perturbation configurations, according to the protocol described in [30]. Prediction of the full set of 256 multi-lesions needed to calculate the neurons' contributions was obtained using projection pursuit regression as the predictor. A cross validation leave-one-out procedure shows that the predictor explains 65%–80% of the data variance depending on the attractant type.

#### Neuronal contribution analysis.

**Figure 3:**
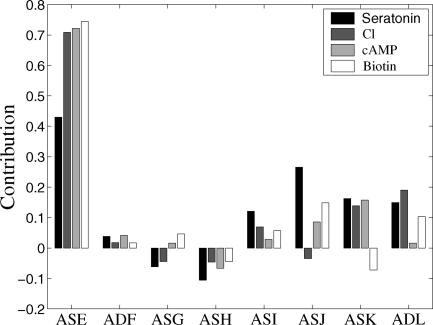
Contributions of the Eight Neuron Pairs to the Different Chemotaxis Attractant Tasks (Normalized Such That Their Sum for Each Attractant Equals One)

Figure 3 displays the contributions of the different neuron pairs to four different attractants tasks (serotonin, chlorine, cAMP, and biotin). As evident, the ASE pair is the most important to chemotaxis across all attractants, in line with the results of Bargmann and Horvitz [30]. However, MPA additionally shows how the importance of all other neurons varies among the different attractants. The processing of the serotonin task is more distributed (the neuronal contributions are more equally spread) across the network than that of the other tasks. A FIN diagram of the relatively simple and localized cAMP task is provided in Protocol S2. Notably, the ASH pair has a negative, inhibitory, contribution to chemotaxis (chemotaxis will be more successful on average if the ASH pair is ablated). This observation is in line with other more recent experimental assays showing that ASH plays a role in mediating *C. elegans* avoidance of toxic chemicals [32]. Interestingly, examining the interaction between these two neuron pairs, ASE and ASH, shows a negative interaction in three attractant tasks, serotonin, biotin, and cAMP (the interaction calculation is described in Materials and Methods). In these tasks, ASE's contribution is suppressed by ASH, i.e., the contribution of ASE is lower when ASH is intact than when it is ablated, and similarly, ASH is suppressed by ASE. Thus, these results lead to the prediction that ASE will be an antagonist of avoidance behavior where ASH is likely to be highly activated.

#### Multiple task analysis.

**Figure 4:**
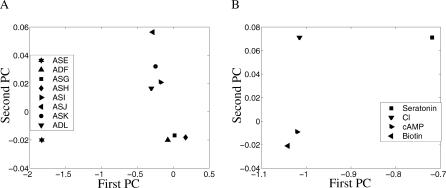
SVD Analysis of the Contribution Matrix Uses the two main principal components of the SVD, which together explain 96% of the data's variance. (A) “Task space,” presenting the projections of the neurons' contribution vectors (column vectors of the contribution matrix) onto the two main principal eigenvectors (PCs) of the task space. (B) *“*Neuron space,” presenting the projections of the tasks' contribution vectors (row vectors of the contribution matrix) onto the two main principal eigenvectors (PCs) of the neuron space.

The contributions of the neuron pairs across the different tasks can be summarized in a contribution matrix, where *C_ij_* in the matrix denotes the contribution of element *j* to task *i* (Table S2). This matrix description permits the utilization of a series of analyses that are not applicable when the results are only summarized in a qualitative manner. Singular value decomposition (SVD), a standard method for dimension reduction previously utilized in various biological applications (e.g., [33]), can be applied to the contribution matrix to reveal both its “neuron space” and its “task space,” identifying similar tasks and similar functional contributions of neurons. Figure 4A presents the results of an SVD of the contribution matrix in the task space. The figure shows four main clusters of the neurons, based on the contributions of different neurons across the attractants (i.e., the column vectors of the contribution matrix in Table S2). The distinct placement of the ASE and ASJ neurons is notable. Neurons participating in each of the clusters {ADF, ASG, ASH} and {ASI, ASK, ADL} have similar functional roles across the investigated attractants. Figure 4B presents the results of SVD in the neuron space. The processing of serotonin chemotaxis is localized very differently than the processing of the other attractants, and the processing of cAMP and biotin is localized in a very similar manner. The similarity between the tasks was already observed by Bargmann and Horvitz [30], although not shown in a rigorous manner. Chemotaxis to chlorine, which was thought to be processed similarly to that of cAMP and biotin [30], is actually processed quite differently.

## Discussion

This paper presents a multi-perturbation analysis of two different biological systems. The analysis reinforces previously known knowledge in a quantitative manner and leads to new insights. The MPA analysis of the PRR system shows that each of the RLCs has a different magnitude of contribution to the PRR process. The FIN analysis gives rise to the hypotheses that there are additional polymerase loading complexes in yeast and that DNA polymerase ζ encoded by *REV3* is probably not the only polymerase involved in PRR. The analysis of *C. elegans*'s chemotaxis provides a more refined picture of the sensory network and rigorously reinforces previous findings.

MPA and FIN are the first methods to our knowledge to harness game theory concepts for the analysis of biological systems. Further work is needed to better adapt these methods to the constraints of biological systems, most notably, the limited depth (i.e., number of concomitantly perturbed elements) of multi-perturbations in biology. However, this is likely to be a very rewarding endeavor, as such multi-perturbation analysis has potentially many applications. The most direct and natural ones are those concerning the analysis of causal perturbation data, e.g., in genetics, using gene silencing with RNA interference. In neuroscience, there is now a new prospect of carrying out experimental perturbation studies using transcranial magnetic stimulation. This technique allows researchers to induce “virtual lesions” in normal subjects performing various cognitive and perceptual tasks [14,15].

Importantly, MPA and FIN are not limited to causal perturbation analysis, where one controls the lesions made. They may well be applied to sets of naturally given multi-perturbations, e.g., by studying the brain localization of cognitive functions from “multi-lesion” data from stroke patients. In summary, multi-perturbation studies are a necessity if one wants to understand the processing of biological networks in a quantitatively causal manner. The methods described in this paper are a harbinger of this new kind of study, offering a novel and rigorous way of making sense out of them.

## Materials and Methods

The basic MPA and FIN analysis methods are described at the beginning of the Results. Here we provide a description of the extension of MPA to a two-dimensional interaction analysis and the details of the FIN algorithm.

### MPA interaction analysis.

In complex systems, the importance of an element may strongly depend on the state (perturbed or intact) of other elements. A higher order description may be necessary to capture these interactions. Such high-dimensional analysis provides further insights into the network's functional organization.

We focus on the description of two-dimensional interactions. A natural definition of the latter is as follows [16]: let


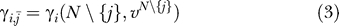


be the Shapley value of element *i* in the subgame of all elements without element *j,* where *v^N^* ^\{*j*}^ is the value function over the set (*N*\{*j*}), which denotes the set *N* without the element *j*. Intuitively, this is the average marginal importance of element *i* when element *j* is perturbed.

Let us now define the coalitional game (*M, v^M^*), where *M* = *N*\{*i, j*} ∪ {(*i, j*)}((*i, j*) is a new compound element composed of both *i* and *j*) and *v^M^* (*S*)*,* for *S* ⊆ *M,* is defined by


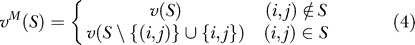


where *v* is the payoff function of the original game with elements *N*. Then γ_(*i, j*)_ = γ_(*i, j*)_(*M, v^M^*), the Shapley value of element (*i, j*), is the average marginal importance of elements *i* and *j* when jointly added to a configuration. The two-dimensional interaction between element *i* and element *j, j ≠ i,* is then defined as


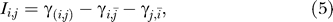


which quantifies how much the average marginal importance of the two elements together is larger (or smaller) than the sum of the average marginal importance of each of them when the other is perturbed. Intuitively, this symmetric definition *(I_i,j_ = I_j,i_)* quantifies the synergistic interaction between elements *i* and *j,* denoting how much “the whole is greater than the sum of its parts.” In cases where the whole is smaller than the sum of its parts, i.e., when the two elements exhibit functional overlap or redundancy, the interaction is negative. Based on the genetic interaction nomenclature of Brendel and Haynes [34], an interaction will be defined as “epistatic” if γ*_i,j¯_* is zero and γ*_i,j_* has a positive contribution, i.e., the intactness of *j* is essential for *i'*s contribution. The Shapley interaction index [35] provides a more general measure for the interaction among players.

### A detailed description of the FIN analysis.

The performance prediction function *F*(*S*) can be uniquely computed as the sum 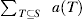 , where *S* denotes the set of intact elements in a given perturbation configuration and the summation goes over all its subsets T [36,37]. The coefficients *a*(*T*) of the summands are the dividends, describing the incremental importance of each summand *T* to the performance being studied. These dividends can be uniquely calculated from the multi-perturbation data (both given and predicted) according to equation 6 (the cardinality of the sets *S* and *T* is denoted by corresponding lower-case letters: *s* = |*S*| and *t* = |*T*|),


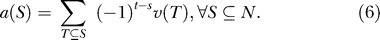


The dividend computation is performed in an iterated manner. It begins from the dividend of the null group, and each iteration computes the dividend (incremental contribution) of subsequently larger, subsuming subsets.

To compute a compact and intelligible approximation of *F,* *F˜* , a greedy heuristic algorithm is employed that retains only the summands *T* with the largest dividends *a*(*T*), while maintaining a predefined level of prediction accuracy. The latter is measured with respect to the performance of the original *F* (by the normalized mean squared error between *F˜* and *F* over all perturbation configurations). The algorithm first selects statistically significant summands (based on a null hypothesis that the dividend magnitude is zero), and then eliminates those with a low magnitude to obtain *F˜* . To visualize *F˜* *,* we construct the FIN diagram. This construction starts with an algebraic simplification, rewriting *F˜* to minimize the number of appearances of each element. This is done by combining clauses and placing elements common to a few summands as multipliers of the weighted summation of the corresponding, residual summands. In the DNA PRR investigation (in the Results), for example, this stage results in the function


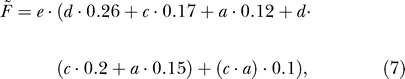


where *a* through *e* are Boolean variables representing the genes, assigned one if the gene is intact and zero if it is knocked out. Based on this simplified representation, we construct the FIN diagram by starting from the function node, *F˜* *,* and connecting it to the variables at the most external level parentheses, assigning weights to the connections according to the corresponding dividend coefficients. This process is recursively repeated by connecting the current leaf nodes on each pathway from the node *F˜* to the next level of elements in the remaining parentheses, until the nodes at the most internal parentheses are connected. The resulting FIN diagram depicts the most important functional pathways (interconnected subsets of elements whose contribution depends on the intactness of the other elements in the pathway) and quantifies their relative importance to the function in hand (see Figure 2).

## Supporting Information

Protocol S1 MPA Robustness—Partial and Noisy Data (47 KB PDF)

Protocol S2 cAMP FIN Diagram (82 KB PDF)

Table S1 Multiple Knockout Data (53 KB PDF)

Table S2 Chemotaxis Contribution Matrix (52 KB PDF)

Text S1 The Axiomatic Basis of the Shapley Value (50 KB PDF)

### Accession Numbers

The SwissProt (http://www.ebi.ac.uk/swissprot/) accession numbers for the *S. cerevisiae* proteins discussed in this paper are Ctf18 (P49956), Elg1 (Q12050), Rad18 (P10862), Rad24 (P32641), and Rev3 (P14284).

## Abbreviations

FIN: functional influence network
MPA: multi-perturbation Shapley value analysis
PRR: post-replication repair
RFC: Replication factor C
RLC: Replication factor C–like complex
SVD: Singular value decomposition
