# Supplementary material for: Quantitative Analysis of Genetic and Neuronal Multi-Perturbation Experiments
Source: PLoS Comput Biol. 2005 Nov 25;1(6):e64. doi: 10.1371/journal.pcbi.0010064 (PMC1289391; doi:10.1371/journal.pcbi.0010064)
Supplement: Protocol S1 — (47 KB PDF) [file pcbi.0010064.sd001.pdf]

Protocol S1: MPA Robustness - Partial and Noisy Data

When applying MPA to biological systems, often not all multi-perturbation experiments are available. In such cases, the predicted MPA variant is used (as described in the main text). To demonstrate the stability of the MPA predicted Shapley value under partial and noisy data we performed two types of analysis; The first examines the experimental neural laser ablation data described in the main text and the second uses simulated data. Analyzing the simulated data has the advantage that we can compare the predicted contributions to the real contributions (whose knowledge we lack in the neural data).

To test the stability of the predicted contributions in the neural ablation analysis, we repeated the process of calculating the predicted contributions using different subsets of the data, according to the leave-one-out scheme, where in each repetition one experiment is left out from the available experimental set. The result of this procedure provides 31 sets of predicted contributions (the dataset includes 31 experiments, and each time we apply the analysis to 30 of them). Figure 1 shows the results of the leave-one-out procedure. Evidently, the predicted contributions are similar to those calculated using all the data, and exhibit stability across the different repeats as noted by the relatively small standard deviations.

In order to rigorously examine the stability and robustness of the MPA under noisy and partial data we used simulated data. In the simulation we assume a system with 8 elements (a,b,c,d,e,f,g,h) that perform a given function F, $F = 0.35a - 0.1(a*e*d) + 0.15(a*e) + 0.05d + 0.15(a*d) + 0.25b - 0.15c + 0.1g + 0.1h + 0.1(g*h)$ (8 elements corresponds to the number of neuron pairs in the laser ablation experiments). The performance F is calculated by assigning one of two states to each of the elements (one if intact and zero if perturbed). Note that F is not a simple linear function, in which case a simple single-perturbation analysis would not be sufficient. Examining F shows that a is the most important element, c has a negative influence on the performance, f has no role at all and g and h are totally symmetric.

Since we have 8 elements in the system there are 256 possible perturbation configurations. Performing these 256 experiments "in-silico" allows us to calculate the exact contribution of each element, which is further used as a benchmark to compare with the MPA outcome of partial and noisy experiments. To examine how sensitive our analysis is to noise, two types of noise were studied.

1. **Measurement Noise**: in each experiment i, the measured performance is $F(i) + whitenoise$, adding noise at the level of 20% (percentage of the performance level when all elements are intact, $F = 1$).

2. **Perturbation Noise**: reflecting the chance that due to technical issues, we did not manage to perturb the elements we planned to, or perturbed elements we did not mean to. To emulate such noise, the state of each element is stochastically perturbed, by randomly flipping its per-

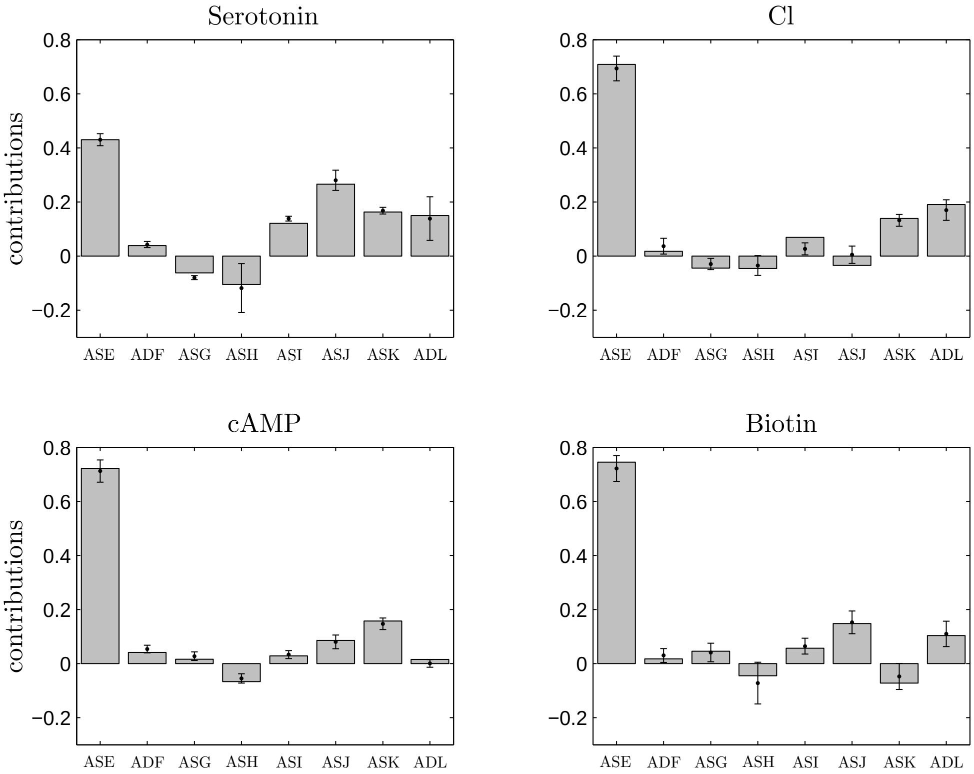

Figure 1: **Leave-one-out contributions:** Predicted contributions of the 8 neuron pairs to the different chemotaxis attractant tasks, portrayed by the gray bars. The black lines describe the mean and standard deviation across different repetitions of the leave-one-out procedure.

turbed/intact state, with a probability of 2.5%. That is, in each experiment there is a chance to actually measure the performance arising from a "wrong" perturbation configuration.

The following figures describe the MPA simulation results, each includes 1000 repeats of adding random noise to the original data and applying MPA to the noisy data. Each MPA repeat returns for each element its contribution, based on two scenarios: 1) All 256 experiments are available, but with noise. 2) Only 31 randomly chosen noisy experiments are available, (out of the 256), to simulate the partial and noisy data scenario. Figures 2 and 3 present the results obtained with the two types of noise. In each of the figures the left pane(a) compares the exact contributions (gray bars) to the contributions found when all 256 experiments are accessible, but with noise added to them (black lines). The right pane(b) shows the predicted contributions (these are based on only 31 randomly chosen experiments with noise), with comparison to the exact contributions. The cross validation score of the two partial datasets show they explain 80.47% of the data variance in the measurement noise case and 92.98% in the perturbation noise case.

To summarize the results, one can see that the main properties of the system are revealed, even under noisy conditions and partial information:

- The predicted contributions are similar to those calculated with full information (all 256 experiments).
- Element a definitely stands out as the most important element and element c as an element with a negative contribution.
- Element g and h show an identical contribution.
- As to element f (the dummy element), while it is not obvious that it has no contribution, a statistical significance test shows that its contribution is not significantly different from zero.

The results show that the predicted contributions are very close to the real ones, even for the small numbers of perturbation configurations used for training (31 out of 256), and exhibits stability across the different runs, as noted by the small standard deviations. The stability of the contributions is explained by the fact that the Shapley value is obtained via averaging over a large number of predictions. Assuming that the predictor is unbiased, prediction errors tend to cancel each other out, resulting in an unbiased predicted Shapley value which is very similar to the real one[1]. These results show that even when the data is noisy and partial the MPA outcome is fairly accurate.

References

[1] Keinan A, Sandbank B, Hilgetag C, Meilijson I, Ruppin E (2005) Axiomatic scalable neurocontroller analysis via the Shapley value. Artificial Life, to appear .

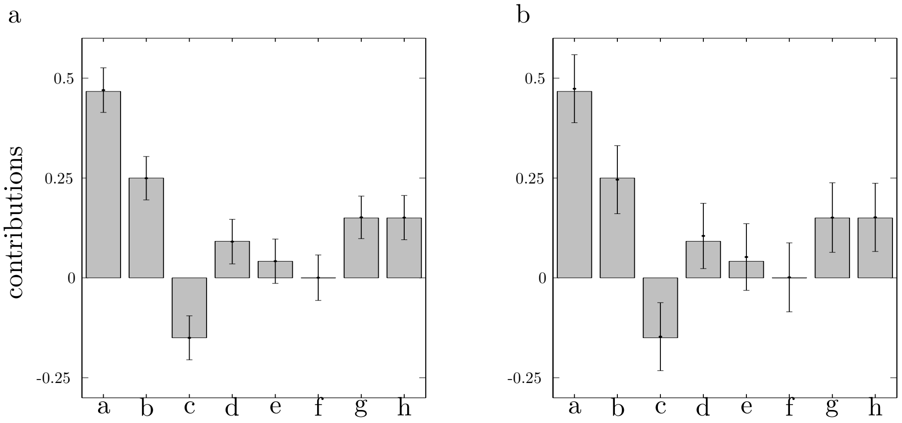

Figure 2: **Measurement noise**: the contributions obtained after adding white noise of 20% to the value of F measured at each multi-perturbation experiment. The left pane(a) shows MPA results on full, noisy data. The right pane (b) shows the MPA results on partial and noisy data. The gray bars describe the real contributions of the 8 elements, the black lines show the mean and standard deviation of the MPA results, repeated 1000 times with random noise. On average, the 31 randomly chosen experiments explain 80.47% of the data variance.

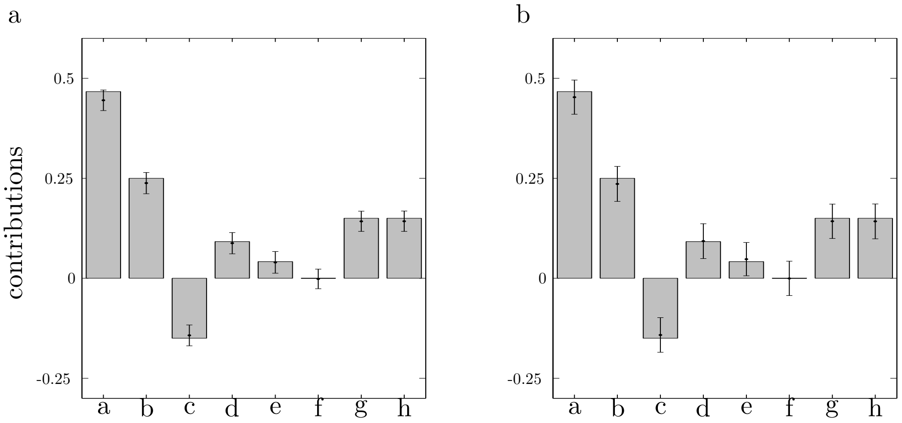

Figure 3: **Perturbation noise:** Each of the eight elements has a chance of 2.5% to be in a different state than expected. The left pane(a) shows MPA results on full, noisy data. The right pane (b) shows the MPA results on partial and noisy data. The gray bars describe the real contributions of the 8 elements, the black lines show the mean and standard deviation of the MPA results, repeated 1000 times with random perturbation noise. On average, the 31 randomly chosen experiments explain 92.98% of the data variance.
