# Supplementary material for: Quantitative Analysis of Genetic and Neuronal Multi-Perturbation Experiments
Source: PLoS Comput Biol. 2005 Nov 25;1(6):e64. doi: 10.1371/journal.pcbi.0010064 (PMC1289391; doi:10.1371/journal.pcbi.0010064)
Supplement: Protocol S2 — (82 KB PDF) [file pcbi.0010064.sd002.pdf]

Protocol S2: cAMP FIN Diagram

Applying the FIN algorithm to analyze the neural ablation data presented in the main text results in a FIN representation for each of the 4 tasks we studied. However, in three out of the four tasks the FIN representation includes many dividends (72, 70 and 103 dividends out of the possible 256 dividends in the Biotin, Cl and Serotonin tasks respectively), resulting in a complex and uninformative FIN diagram[1]. In the cAMP task, however, the FIN compact representation includes only 5 dividends which results in a clear and informative diagram presented in Figure 1.

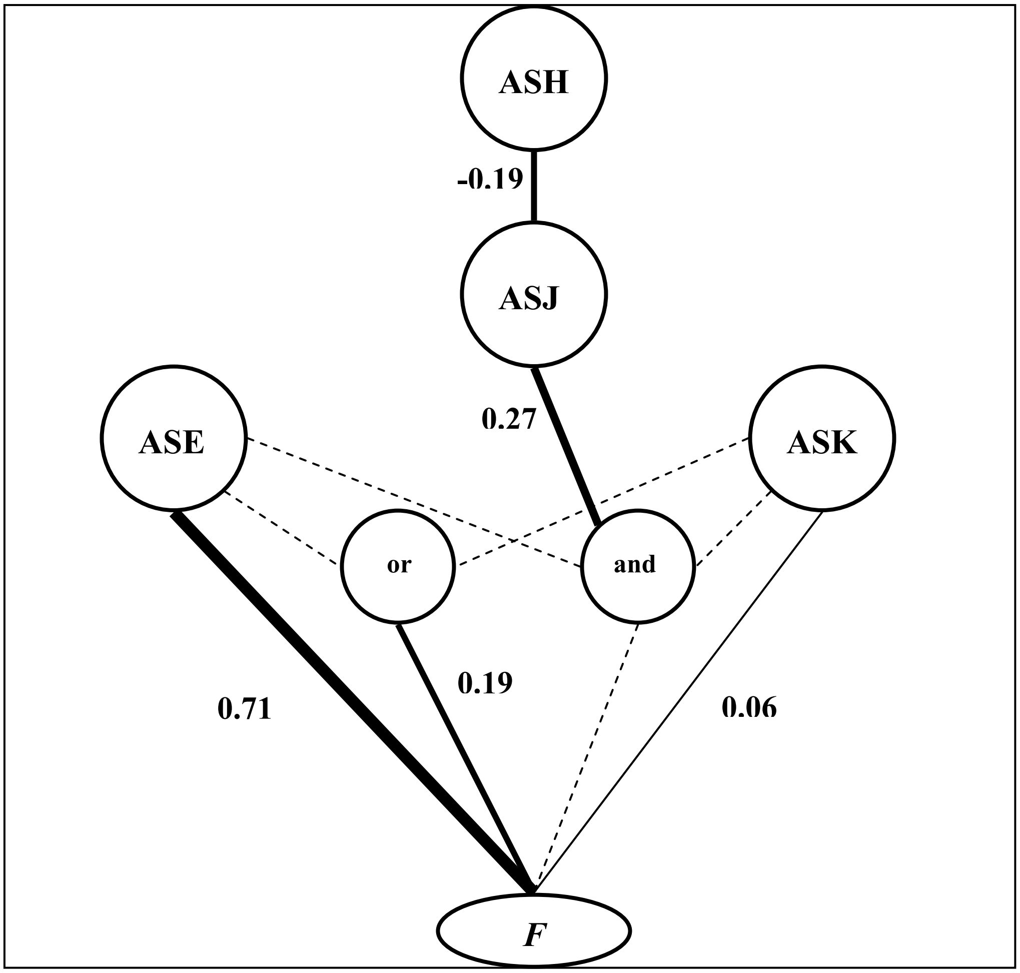

Figure 1: **The FIN Diagram of cAMP Chemotaxis:** the figure visualizes the compact performance function *F=0.9ASE+0.25ASK+ASE·ASK(-0.19+ASJ(0.27-0.19ASH))*. The neuron pairs are represented as binary nodes, whose state is determined according to the state of the corresponding neurons in a given perturbation configuration, intact or ablated. As in the FIN diagram in the main text the nodes are connected with edges, their weight representing the functional influence between the two endpoint nodes (the width of the edge is proportional

---

[1] The failure to find a simple FIN representation may arise in principal from two main reasons: 1) There is no compact function underlying the behavior studied or 2) The algorithm was unable to reveal an existing compact functional representation. New versions of the FIN algorithm are currently under development to address the latter case.

to its weight). However, in addition dashed lines represent connectivity edges which define logic relations and have no weights. Given a perturbation configuration, the expected performance level *F* can be calculated by summing up the weights on the edges between intact nodes which form a connected component with the function node *F*, including the connectivity edges and considering the Boolean relations of the respective nodes. E.g., if only ASE and ASJ are intact, the intact nodes used in the performance prediction will be the ASE, ASJ and the 'or' (ASE or ASK) nodes, the 'and' node will be ignored (it requires ASE **and** ASK to be intact), in this case the resulting connected subgraph of *F* will include only two edges and predict a performance level of 0.9 (0.71+0.19).

The cAMP FIN includes only 4 neuron pairs out of the optional 8, and explains the experimental data with an accuracy of 88% (normalized MSE:0.12). Its FIN diagram proposes the following insights:

- ASE by itself is liable to 90% of the chemotaxis functionality, that is, if all other neurons are ablated and only ASE remains, the performance level is reduced by 10%. This notation of liability is conceptually different from the MPA outcome that shows the ASE contribution is 73%. For example, in a two elements system were the two elements are redundant, each may be liable to 100% of the functionality but the relative contribution of each element is 50%.
- ASK and ASE are redundant in one of the functional pathways (19%), however, in another functional pathway with ASJ and ASH, ASE and ASK are both required.
- The 4 absent neuron pairs (ADF, ASG, ASI, ADF) play a negligible role in the main functional subnetworks responsible for cAMP chemotaxis.
