# Supplementary material for: Quantitative Analysis of Genetic and Neuronal Multi-Perturbation Experiments
Source: PLoS Comput Biol. 2005 Nov 25;1(6):e64. doi: 10.1371/journal.pcbi.0010064 (PMC1289391; doi:10.1371/journal.pcbi.0010064)
Supplement: Table S1 — (53 KB PDF) [file pcbi.0010064.st001.pdf]

Table S1: Genetic Multiple Knockout Data, post replication repair in yeast *S. cerevisiae*.

The table presents the genetic multi-knockout experiments performed. Each row summarizes one specific experiment, in which the different genes (columns) are either knocked-out (0) or intact (1). The performance measure of each mutant is the percent of colonies that survive the UV radiation.

ELG1	CTF18	RAD24	REV3	RAD18	Performance
1	1	1	1	1	85.7
0	1	1	1	1	69.7
1	0	1	1	1	69.4
1	1	0	1	1	54.7
1	1	1	0	1	28.5
0	0	1	1	1	33.3
0	1	0	1	1	21
0	1	1	0	1	24.5
1	0	0	1	1	51.9
1	0	1	0	1	39.5
1	1	0	0	1	1.6
0	0	0	1	1	16.4
0	0	1	0	1	22.3
0	1	0	0	0	0.25
1	1	1	1	0	0.143
0	1	1	1	0	0.09
1	0	1	1	0	0.11
1	1	0	1	0	0.026
0	0	1	1	0	0.05
1	0	0	1	0	0.0025
1	1	1	0	0	0.13
