# Supplementary material for: Quantitative Analysis of Genetic and Neuronal Multi-Perturbation Experiments
Source: PLoS Comput Biol. 2005 Nov 25;1(6):e64. doi: 10.1371/journal.pcbi.0010064 (PMC1289391; doi:10.1371/journal.pcbi.0010064)
Supplement: Table S2 — (52 KB PDF) [file pcbi.0010064.st002.pdf]

Table S2: Chemotaxis Contribution Matrix

The matrix portrays the contributions of the 8 neuron pairs across the different attractant tasks, the contributions are normalized such that their sum in each task (row of the matrix) equals to one. C_{ij} in the matrix denotes the contribution of element j to task i. Figure 3 in the main text shows a graphic representation of the contribution matrix.

	ASE	ADF	ASG	ASH	ASI	ASJ	ASK	ADL
Serotonin	0.4300	0.0383	-0.0619	-0.1055	0.1210	0.2658	0.1628	0.1496
Cl	0.7084	0.0182	-0.0442	-0.0460	0.0693	-0.0346	0.1387	0.1902
cAMP	0.7219	0.0415	0.0159	-0.0666	0.0285	0.0857	0.1573	0.0158
Biotin	0.7445	00172	0.0459	-0.0446	0.0569	0.1485	-0.0721	0.1036
