# Supplementary material for: Quantitative Analysis of Genetic and Neuronal Multi-Perturbation Experiments
Source: PLoS Comput Biol. 2005 Nov 25;1(6):e64. doi: 10.1371/journal.pcbi.0010064 (PMC1289391; doi:10.1371/journal.pcbi.0010064)
Supplement: Text S1 — (50 KB PDF) [file pcbi.0010064.sd003.pdf]

# Text S1: The Axiomatic Basis of the Shapley Value

Let player $i$ be a *null player* in $v$ if $\Delta_i(S) = 0$ for every coalition $S$ $(i \notin S)$. Players $i$ and $j$ are *interchangeable* in $v$ if $\Delta_i(S) = \Delta_j(S)$ for every coalition $S$ that contains neither $i$ nor $j$. The Shapley value is the only efficient value that satisfies the three following axioms:

**Axiom 1** *(Symmetry) If $i$ and $j$ are interchangeable in game $v$ then $\gamma_i(v) = \gamma_j(v)$.*

Intuitively, this axiom states that the value should not be affected by a mere change in the players' "names".

**Axiom 2** *(Null player property) If $i$ is a null player in game $v$ then $\gamma_i(v) = 0$.*

This axiom sets the baseline of the value to be zero for a player whose marginal importance is always zero.

**Axiom 3** *(Additivity) For any two games $v$ and $w$ on a set $N$ of players, $\gamma_i(v+w) = \gamma_i(v)+\gamma_i(w)$ for all $i \in N$, where $v+w$ is the game defined by $(v+w)(S) = v(S) + w(S)$.*

This last axiom constrains the value to be consistent in the space of all games.
